# The Effects of GABA-Rich Adzuki Beans on Glycolipid Metabolism, as Well as Intestinal Flora, in Type 2 Diabetic Mice

**DOI:** 10.3389/fnut.2022.849529

**Published:** 2022-02-14

**Authors:** Aiwu Zhang, Xiujie Jiang, Yunfei Ge, Qingpeng Xu, Zhijiang Li, Huacheng Tang, Dongmei Cao, Dongjie Zhang

**Affiliations:** ^1^College of Food Science, Heilongjiang Bayi Agricultural University, Daqing, China; ^2^National Coarse Cereals Engineering Research Center, Heilongjiang Bayi Agricultural University, Daqing, China; ^3^Department of Marine Food Science and Technology, Gangneung-Wonju National University, Gangneung, South Korea

**Keywords:** type 2 diabetes, glycolipid metabolism, gut microbiota, GABA, sprouted adzuki beans

## Abstract

**Objectives:**

In this study, the effects of γ-aminobutyric acid (GABA)-rich sprouted adzuki beans on the glycolipid metabolism and gastrointestinal health were investigated in mice with type 2 diabetes mellitus (T2DM).

**Methods:**

Mice with T2DM were subjected to dietary intervention with different doses of GABA-rich sprouted adzuki beans for 6 consecutive weeks, during which growth indicators, glycolipid metabolism, and the composition and diversity of the gut microbiota changes were observed.

**Results:**

A high dietary intake of GABA-rich sprouted adzuki beans had a preventive effect against weight gain, significantly reduced serum levels of FBG, TG, and TC. Additionally, high dietary intake of GABA-rich sprouted adzuki beans increased the abundances of Firmicutes, Bacteroidetes, Verrucomicrobia, and Akkermansia, leading to a shift in the structure of the gut microbiota toward the dominance of probiotics with regulatory effects on glycolipid metabolism.

**Conclusions:**

GABA-rich sprouted adzuki beans can effectively control the bodyweight of mice with T2DM, maintain a balanced blood glucose level, improve glycolipid metabolism and the changes in the microbiota may mediate the anti-diabetic effect of sprouted adzuki beans.

## Introduction

Diabetes is a metabolic disease, the pathophysiology is linked to insulin levels in the body and the body's ability to use insulin. Type 1 diabetes is characterized by a complete lack of insulin, whereas type 2 diabetes is characterized by peripheral tissue resistance to the effects of insulin (1). Type 2 diabetes (T2DM) is a chronic metabolic disease characterized by abnormal insulin secretion or defective insulin action. It is characterized by impaired glucose metabolism disorders, local hyperglycemia, as well as impaired lipid and protein metabolism (2). According to data issued by the International Diabetes Federation (IDF), the global diabetes population reached 463 million in 2019, with nearly 90 percent of them suffering from T2DM. Furthermore, if certain interventions are not implemented, the number of diabetic patients will exceed 700 million by 2045 (3). The rapid increase in the incidence of T2DM has become a global health issue, largely due to its difficulty to treat and complications (4).

Dietary regulation is the cornerstone of the prevention and control of diabetes, as diet contributes to its onset and progression (5). Coarse cereals are hypoglycaemic foods with various functional components, among which γ-aminobutyric acid (GABA) is a non-protein amino acid that has become a new resource in the food industry owing to its multiple physiological functions, including its ability to lower blood pressure, improve sleep quality, and protect against T2DM (6, 7). GABA can be selectively synthesized and secreted in pancreatic β-cells (8). It can depolarize the membrane potential of islet cells by activating GABA receptors (GABAbR) on the cell membrane to enhance insulin secretion and maintain glucose homeostasis (9). For instance, previous research confirmed that GABA can alleviate pancreatitis and autoimmune β-cell damage (10). Additionally, GABA can induce the transformation of α-cells to β-cells (11) and mediate the regeneration of β-like cells (12). These findings have provided unprecedented hope for the treatment of diabetes.

Adzuki bean contains a high amount of sodium glutamate (MSG), which is a good source of GABA, an earlier study has shown that germination treatment can increase GABA content in the bean (13), and dietary supplementation with GABA or GABA-rich foods is a common strategy for the treatment of T2DM. GABA-rich brown rice improves the blood glucose level of rats with T2DM (14) and has protective effects against liver and pancreatic injury (15). The dietary intake of sprouted black rice also helps to prevent and control T2DM (16). Additionally, GABA-rich yogurt can improve insulin sensitivity and islet cell morphology in mice with T2DM (17). It is interesting to note that GABA supplementation significantly alters gut microbial diversity in rats (18). Therefore, we fed mice with streptozotocin (STZ)-induced T2DM sprouted adzuki beans subjected to GABA enrichment via vacuum impregnation to evaluate its effects on growth and gastrointestinal health based on blood glucose levels, blood lipids, and the gut microbiota composition. The goal of this study is to discover the regulatory effects of dietary intervention with GABA-rich sprouted adzuki beans on the glycolipid metabolism and gut microbiota in T2DM mice, providing a technical guide for the development of GABA-rich functional, sprouted foods capable of regulating glycolipid metabolism.

## Materials and Methods

### Materials and Chemicals

The variety of Adzuki beans are pearl red that were obtained from a local company (Ruizefeng Technology Co., Ltd., Daqing, China) in 2021. The γ-aminobutyric acid standard and STZ were obtained from Sigma-Aldrich (St. Louis, MO, USA). Kits for fasting blood glucose (FBG), total cholesterol (TC), triglycerides (TG), aspartate aminotransferase (AST), and alanine aminotransferase (ALT) tests were obtained from Roche Diagnostics Co., Ltd. (Shanghai, China). The kit of fasting insulin was obtained from Yisan Biotechnology Co., Ltd (Beijing, China). Other reagents (analytical grade) were obtained from Damao Chemical Reagent Co., Ltd (Tianjin, China).

### Adzuki Bean Sprouting Preparation

Adzuki beans were washed with water before being immersed for 15 min in 1.0 percent sodium hypochlorite (NaClO) and washed three times with filtered water, followed by 16 h in 4 volumes of 0.4 mmol/L CaCl_2_ (pH 5) at 35°C. The adzuki beans were then rinsed with deionized water and evenly spread on a germination plate wrapped in four layers of gauze. After being covered with wet gauze, the adzuki beans were spray distilled water and MSG, which were placed in a vacuum drying oven and nurtured for 16 h at 31°C and −0.1 MPa. A single-factor orthogonal test was used to determine the optimal soaking and germination conditions, and the optimal soaking temperature, soaking time, and pH conditions were 35°C, 16 h, and 5, respectively. Furthermore, the optimal germination conditions for optimal GABA enrichment were 48 h, 1.99 mg/ml MSG concentration, 31.49°C germination temperature, and 15.83 h vacuum time. The germinated adzuki beans were then divided into two parts: one was freeze-dried and used to measure GABA levels (our recent article (13) reported the preparation process of germinated adzuki beans and the optimization of GABA content) and subsequent animal experiments.

### Laboratory Animals, Model Construction, Groups, and Doses

Sixty-four 6-week-old C57BL/6J male mice (male) weighing 18–22 g were ordered from professional animal breeding institutions (Changchun Yisi Experimental Animal Technology Co., Ltd., Changchun, China). This company's license number is SCXK (Ji)2018-0007). Every five mice were placed in a cage and kept in a humidified environment with a humidity of 45–50% and a temperature of 20–25°C. For 1 week before the start of the experiment, the animals were fed in an adaptive manner, during which their emotional state was carefully observed and their daily food intake was recorded to provide a reference for determining the intervention dose for subsequent experiments. All experiments were conducted following the guidelines and acceptance of the Heilongjiang Bayi Agricultural University's Animal Use Ethics Committee.

Choose healthy C57BL/6J mice and divide them into two groups: normal control (*n* = 8) and high-fat diet (HFD) + STZ induced diabetes (model) (*n* = 56). During the modeling period, mice were fed normal chow, which in the normal group, while a high-fat diet (TP23300, containing 60% fat) (TROPHIC Animal Feed High-Tech Co. Ltd., Nantong, China) was given to mice in the model group for 4 weeks. The mice in the model group were injected intraperitoneally with STZ at a concentration of 50 mg/kg after 12 h of fasting and water deprivation. The mice in the normal control group received the same dose of Citrate buffer intraperitoneally. The level of frontal FBG in mice was measured after 3 days of STZ injection. When the FBG level in the model group surpassed 11.1 mmol/L for 3 days in a row, the model has been successfully constructed (19). Continue the experiment with the successfully modeled mice.

Successfully constructed T2DM mice models were assigned to some groups at random, and the animal grouping and feeding dose are shown in Table 1. Mice in the TF1, TF2, TF3, and B groups were subjected to dietary intervention with different doses of sprouted adzuki beans in place of HFD. The M group was provided HFD. The C group was fed basic feed, while mice in the TG and TS groups were treated with HFD + pharmacological intervention. Supplementary Figure 1 depicts the specific experimental design.

**Table 1:** Animal experiment group.

**Group**	**Repeat (number)**	**Feed**	**Treatment dose**	**Time (week)**
C	8	Basic feed	-	6
M	8	HFD	-	6
TF1	8	HFD + low-dose GABA-rich sprouted adzuki beans	15 g/100 g	6
TF2	8	HFD + medium-dose GABA-richsprouted adzuki beans	25 g/100 g	6
TF3	8	HFD + high-dose GABA-rich sprouted adzuki beans	35 g/100 g	6
B	8	HFD+ adzuki beans	35 g/100 g	6
TG	8	HFD + GABA	0.1 g/kg	6
TS	8	HFD + metformin	0.1 g/kg	6

Every 2 weeks during the dietary intervention, the body weight and FBG levels of each group of mice were measured. The mice were fasted for 12 h to drink water, anesthetized with sodium pentobarbital, dissected and collected blood, maintained at 25°C for 30 min and centrifuged at 3,000 rpm for 10 min at 4°C to collect the sample serum.

### Measurement of Biochemical Markers for Glycolipid Metabolism

After 5 weeks of dietary and pharmacological interventions, serum levels of fasting insulin (FINS) were measured as per instructions provided in the kit. Serum levels of TC, TG, FBG, ALT, and AST were evaluated using the Roche Cobas c702 Automated Biochemical Analyzer (Jiabang Electronic Technology Co., Ltd., Guangzhou China).

### Histopathological Analysis

After dietary and pharmacological interventions, mice were dissected to harvest a small fragment of liver and caecal tissues, which were immediately fixed in 4% paraformaldehyde and sent to Wuhan Boerfu Biotechnology Co., Ltd. (Wuhan, China) for hematoxylin-eosin (HE) staining. The morphology of the liver and caecal tissues were observed using the BA210 microscope-equipped camera (Maichi Optoelectronics Technology Co., Ltd., Chongqing China).

### Determination of the Gut Microbiota Composition

A total of 200 mg of cecal contents was added to a centrifuge tube with 1 mL of 70% ethyl alcohol, vibrated to mix, and centrifuged at room temperature for 3 min at 10,000 rpm. After removing the supernatant, 1 mL of PBS was added, mixed, and centrifuged for 3 min at 10,000 rpm at room temperature. After removing the supernatant, the tubes were inverted for 1 min, or until all remaining liquid was removed. To remove any remaining ethyl alcohol, sample tubes were dried at 55°C for 10 min. Bacterial DNA was extracted from cecal contents using a DNA kit (Nanjing Jiancheng Bioengineering Research Institute), and extraction was carried out according to the manufacturer's protocol. Microbial diversity was determined by sequencing the V3-V4 region of 16S rDNA by Beijing Biomarker Technologies Co., Ltd (Beijing, China).

### Statistical Analysis

Microsoft Excel and SPSS software were used for statistical analysis. Origin software was used for image processing. All data were collected in triplicate and the average value was used for analysis.

## Results

### The Effects of Various Dosages of GABA-Rich Sprouted Adzuki Beans on the Body Weight and FBG Level of Mice With T2DM

**Figure 1:**
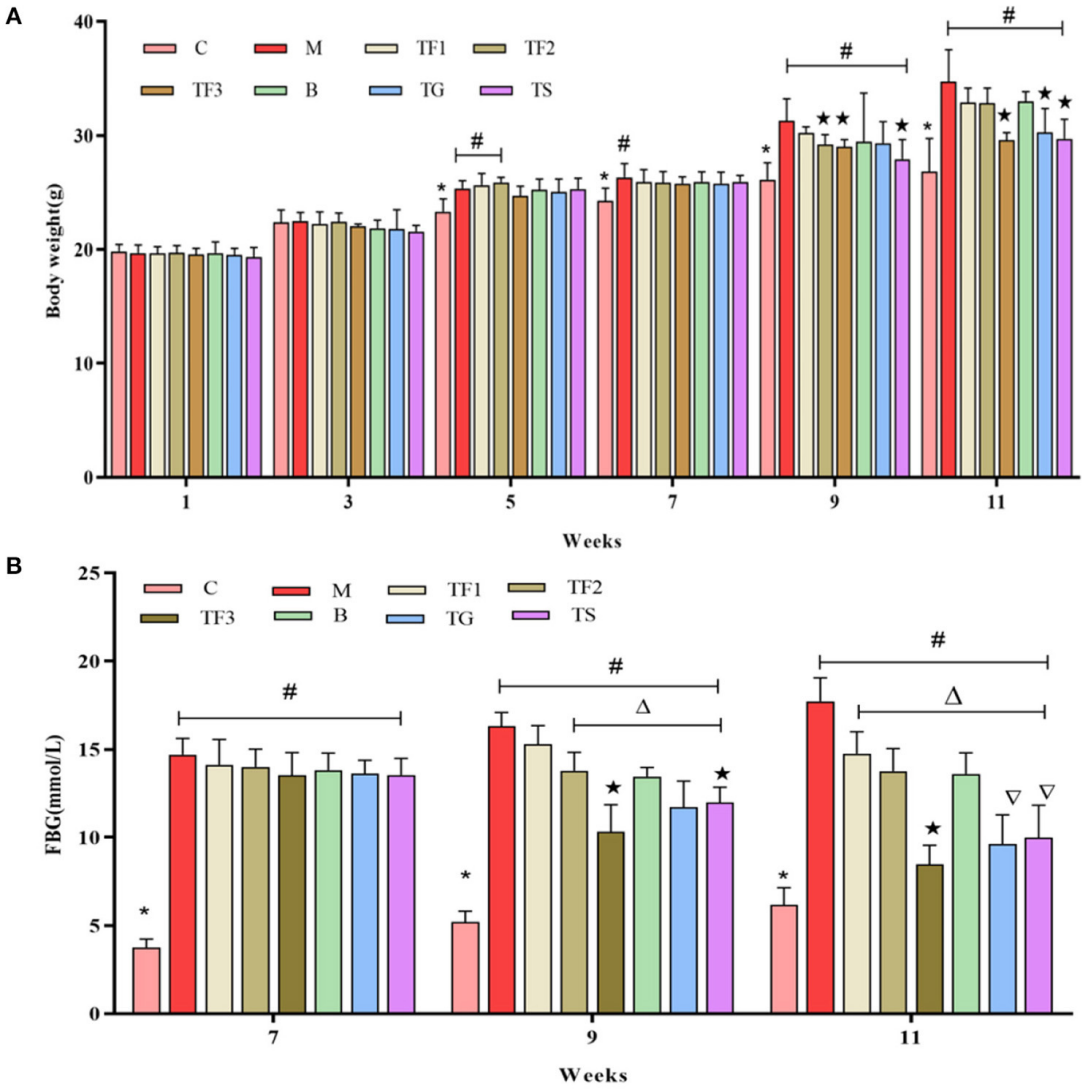
Effect of GABA-rich sprouted adzuki beans on the body weight (BW) and FBG level of mice with T2DM. The values represent the mean ± SEM (*n* ≥ 5); **(A)** BW of mice at different time points; **(B)** FBG levels of mice during dietary intervention; values with different symbols differ substantially (*p* < 0.05) from each other.

At week 11, BW was considerably higher in the M group than in the C group and considerably lower in the TF3, TG, and TS groups than in the model (T2DM) group (*p* < 0.05), implying that dietary intervention with GABA-rich sprouted adzuki beans and oral administration of GABA (TG) and metformin (TS) can reduce BW in mice with T2DM, with a preventive effect against weight gain (Figure 1A). At week 7, all mice with diabetes had significantly higher FBG levels than mice in the C group (*p* **<** 0.05), as shown in Figure 1B. After 6 weeks of intervention with GABA-rich sprouted adzuki beans, GABA, and metformin (at week 11 of the experiment), mice in all of the treatment groups had significantly lower FBG levels than those of mice in the M group, in particular, the TF3, TG, and TS groups displayed 50.98, 44.13, and 42.04% decreases in FBG levels. These results suggested that GABA-rich sprouted adzuki beans yield the best performance with respect to the maintenance of a balanced blood glucose level, followed by GABA and metformin. These findings are consistent with those of previous studies (20).

### The Effects of Various Dosages of GABA-Rich Sprouted Adzuki Beans on Blood Glucose Levels in Mice With T2DM

**Figure 2:**
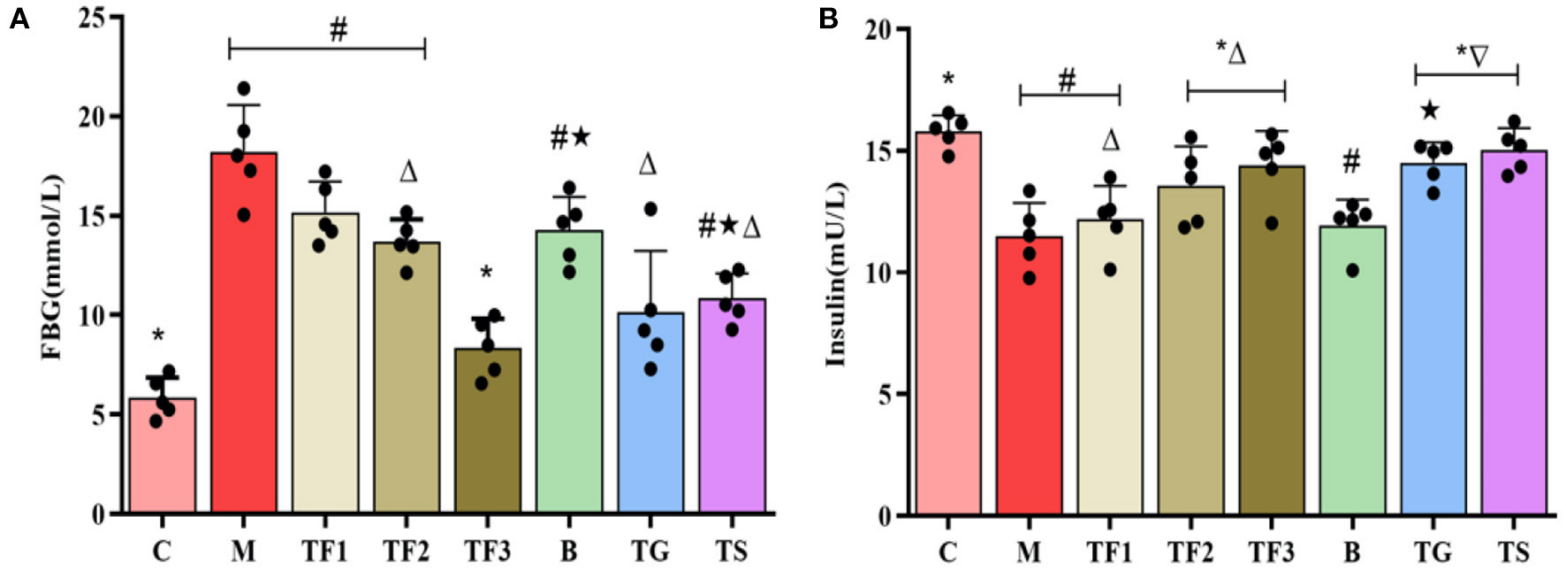
Effects of GABA-rich sprouted adzuki beans on serum markers of glucose metabolism in mice with T2DM. The values represent the mean ± SEM (*n* ≥ 5); **(A)** FBG; **(B)** INS. Values with different symbols differ substantially (*p* < 0.05) from each other.

Abnormalities in serum biochemical markers are closely associated with T2DM, among which FBG and FINS levels are important markers of glucose metabolism and islet function. The serum FBG test in this study (Figure 2A) revealed that after 6 weeks of diet intervention, in TF1, TF2, and TF3 groups, the blood glucose levels of mice were lower than those of the M group. In particular, the TF3 group showed the most significant decline in blood glucose levels (*p* < 0.05). A FINS test (Figure 2B) revealed that the TF3, TG, and TS groups had slightly higher INS levels than those of the M group (*p* > 0.05).

### The Effects of Various Dosages of GABA-Rich Sprouted Adzuki Beans on Blood Lipids and Markers of Liver and Kidney Functions in Mice With T2DM

**Figure 3:**
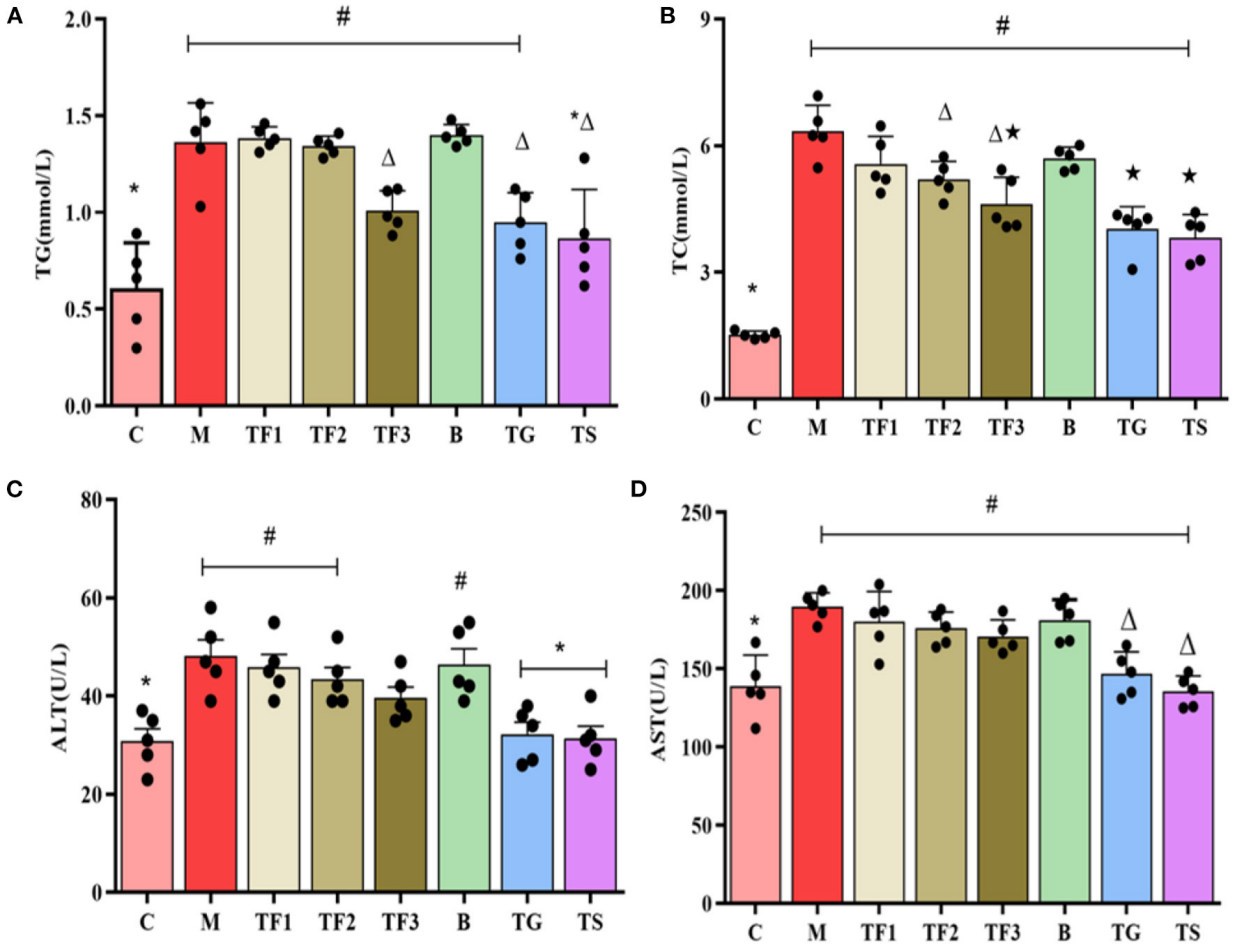
The effects of various dosages of GABA-rich sprouted adzuki beans on blood lipid levels and liver and kidney function markers in mice with T2DM. The values represent the mean ± SEM (*n* = 5); blood lipids: **(A)** TG, **(B)** TC; markers of liver function: **(C)** ALT, **(D)** AST. Values with different symbols differ substantially (*p* < 0.05) from each other.

As shown in Figures 3A,B, various dosages of GABA-rich sprouted adzuki beans can reduce the serum levels of TG and TC to varying degrees in mice with T2DM. The TG and TC levels of mice in the TF3 group were 32.9 and 48.8% lower than those of mice in the M group, respectively. Mice in the treatment groups had significantly higher serum ALT and AST levels than mice in the control group (C) (*p* < 0.05). The ALT and AST serum levels in the TF3 group were slightly lower than those in the M group (*p* < 0.05) (Figures 3C,D). The magnitudes of increases in blood concentrations of ALT and AST were positively correlated with the severity of hepatocellular damage. Mice with T2DM showed severe liver injury and this was alleviated by the high-dose intake of GABA-rich sprouted adzuki beans.

### Pathological Analysis of the Liver and Intestine in Mice With T2DM

**Figure 4:**
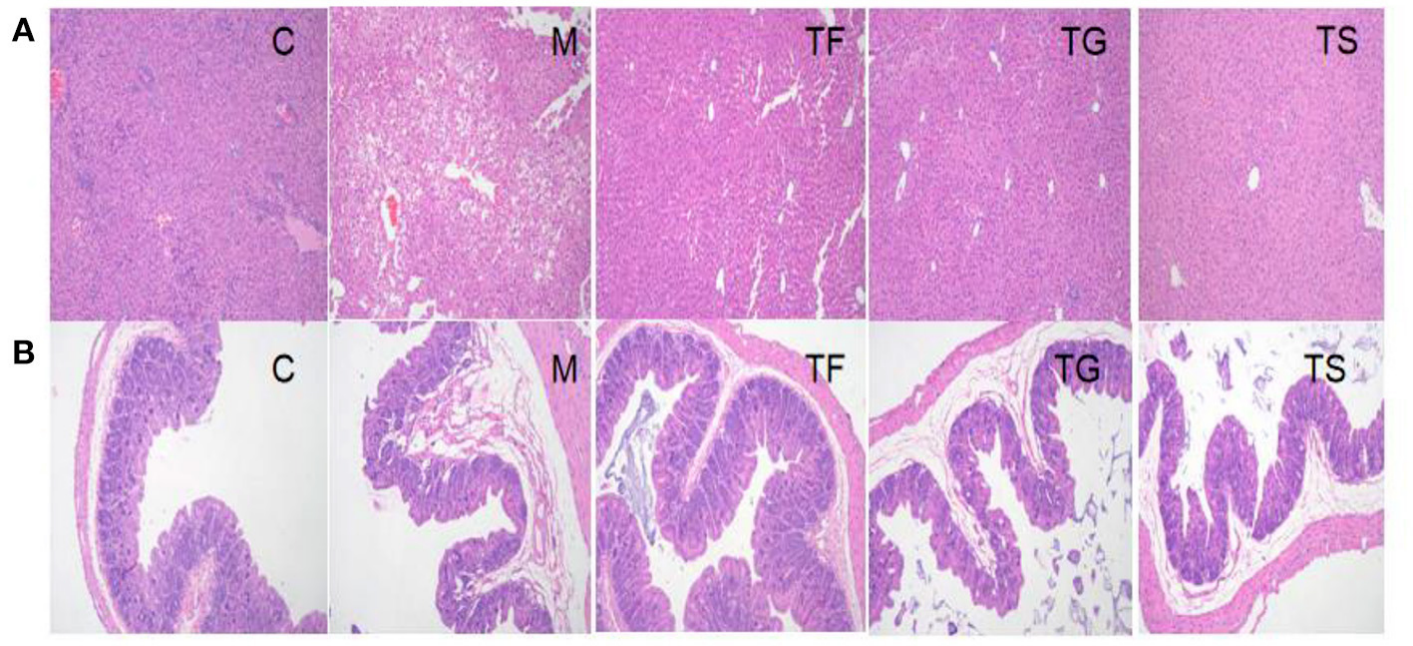
Pathological analysis of **(A)** liver and **(B)** caecal tissues from mice in different groups by HE staining (×100). C: normal control group; M: HFD+ STZ-induced diabetes (model) group; TF: HFD + high-dose GABA-rich sprouted adzuki beans (35 g/100 g) group; TG: HFD + GABA intervention (0.1 g/kg) group; TS: HFD + metformin (0.1 g/kg) group.

Based on serum biochemical tests, five representative groups, i.e., C, M, TF, TG, and TS groups, were selected for a pathological analysis and gut microbiota profiling. The HE staining results for liver and caecal tissues from mice in these five groups are shown in Figure 4.

Figure 4A shows that mice in the TF groups had regularly arranged hepatocytes with no hyperemia and a reduced number of vacuoles as compared with mice in the M group; however, there were local lipid droplets of various sizes. Mice in both the TG and TS groups had morphologically normal liver tissues; however, the number of vacuoles was higher in the former than the latter. Taken together, the liver tissue of the TS group was most similar in morphology to that of the C group, followed by the TG and TF groups. As shown in Figure 4B, some of the intestinal villi fell off and accumulated along with substances in the intestinal lumen of the M group, with some degenerated and necrotic epithelial cells accompanied by inflammatory cell infiltration. Additionally, the intestinal glands degenerated and showed the formation of vacuoles. However, these pathological features were improved to some extent following interventions with TF, TG, and TS.

### Effects of GABA-Rich Sprouted Adzuki Beans on Microbial Diversity in the Caecum of Mice With T2DM

**Figure 5:**
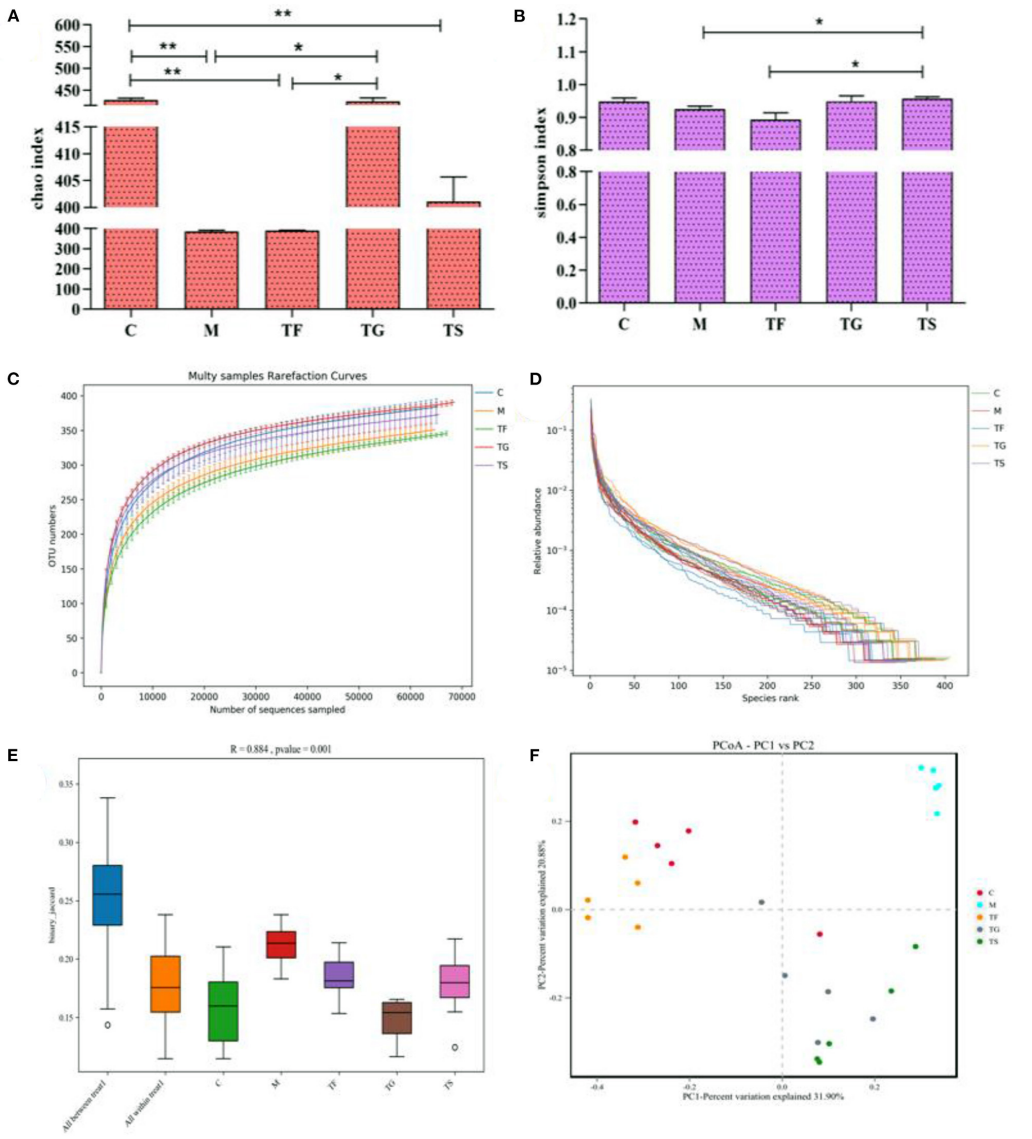
Effects of different treatments on gut microbial diversity in mice. **(A)** Chao index; **(B)** Simpson index; **(C)** Rarefaction curves of OTUs; **(D)** Rank abundance curves; **(E)** Boxplot of β-diversity distances; and **(F)** Between-group principal component analysis (PCA) plots. Results are expressed as means and standard deviations of five samples for each group (25 total samples). The values with ^**^ symbols indicate that they differ substantially (*p* < 0.05) from each other.

The Chao index, as shown in Figure 5A, reflects the total number of microbial species; a higher value indicates a higher number of microbial species. The Simpson index is inversely proportional to species richness and evenness. In the TF group, the Chao index for the gut microbiota was higher than M group, but in the M group it was lower than TG, TF, and C groups; the TG group had the highest Chao index, which was not significantly different from that in the positive control group (Figure 5A). Additionally, the Simpson index was lowest in the TF group (Figure 5B). The rarefaction curve of OTUs (Figure 5C) and the rank abundance curve (Figure 5D) for each group showed that in the TF group the α-diversity of microbiota was lower than C group and higher than that in the model group. The rarefaction curve for each group tended to reach a plateau at a sequencing depth of 10,000 reads, indicating a sufficient sequencing depth. The boxplot of β-diversity distances visually reflects the similarity between samples within the same group and differences between groups. As shown in Figure 5E, there were substantial differences in the structure of the microbial community among the groups (*p* < 0.01). PC1 described a sizable proportion of the variance (46.07%), while PC3 described a small proportion of the variance (9.76%) (Figure 5F), indicating that there was high similarity within groups and apparent divergence between groups.

### Effects of GABA-Rich Sprouted Adzuki Beans on the Microbial Composition in the Caecum of Mice With T2DM

**Figure 6:**
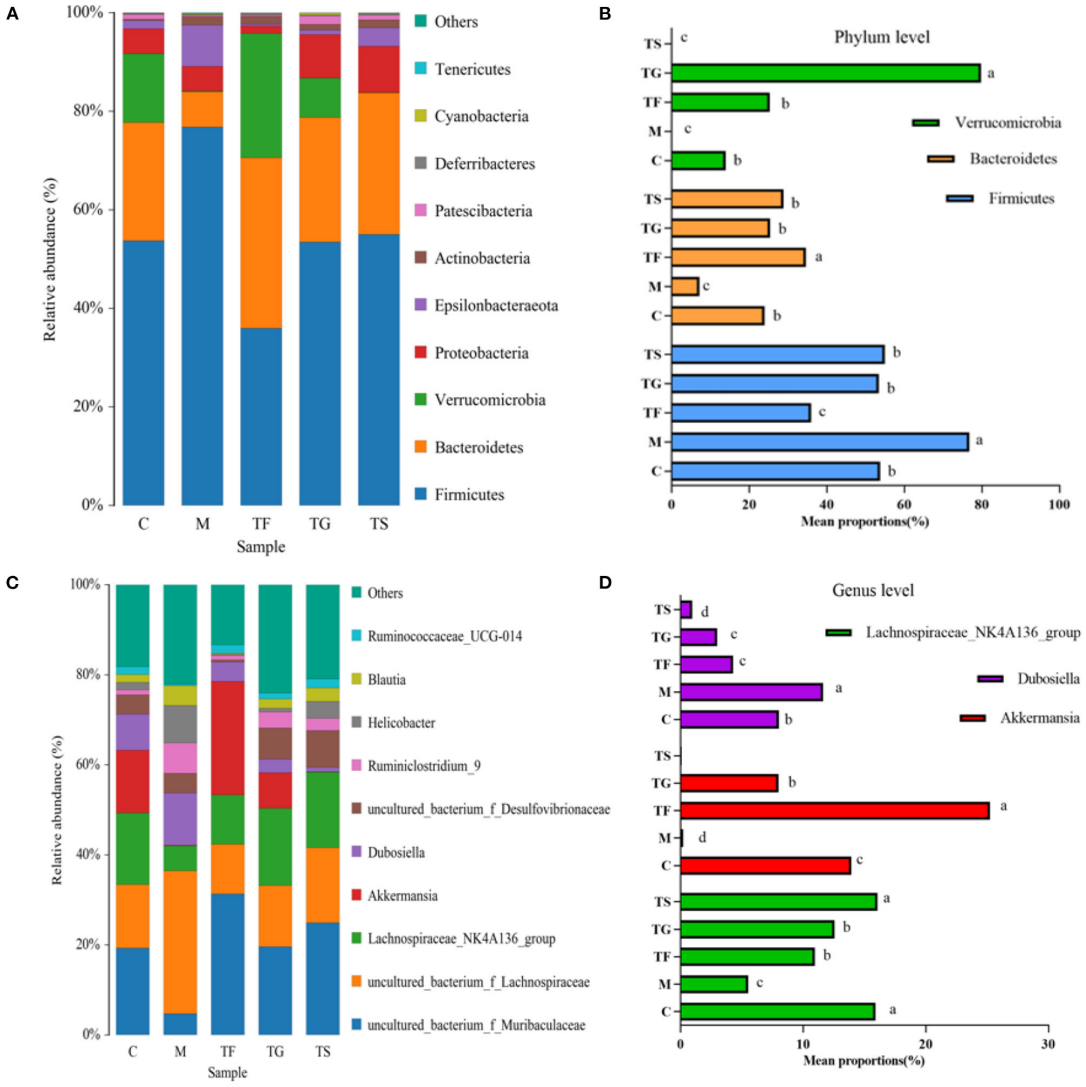
Effects of GABA-rich sprouted adzuki beans on the microbial composition of the caecum in mice with T2DM. **(A)** Relative abundances of microbial taxa at the phylum level. **(B)** Comparative analysis of the top three phyla with the highest relative abundances. **(C)** Relative abundances of microbial taxa at the genus level. **(D)** Comparative analysis of the top three genera (excluding non-annotated OTUs at the genus level) with the highest relative abundances. The values with different symbols indicate that they differ substantially (*p* < 0.05) from each other.

As summarized in Figure 6, there were six dominant phyla in the caecum of mice, i.e., Firmicutes, Bacteroidetes, Verrucomicrobia, Proteobacteria, Epsilonbacteraeota, and Actinobacteria. In the M group and the TF group, the relative abundances of Firmicutes at the phylum level were 42.78% higher and 33.13% lower than the C group, respectively. However, the relative abundances of Firmicutes in the TG and TS groups did not vary from those in the C group (Figures 6A,B). The relative abundance of Bacteroidetes was highest in the TF group (34.62%) and lowest in the M group (7.14%). The relative abundance of Verrucomicrobia in the TG group was obviously higher than that in the C group, while the TS group and the M group had an obviously lower relative abundance of Verrucomicrobia than that in the C group, accounting for 0.2 and 0.07% of the microbiota, respectively. There was no notable disparity in the abundance of Verrucomicrobia between the TF and C groups. The genus-level differential abundance analysis of the gut microbiota revealed that the TF group had a significantly higher relative abundance of Akkermansia (*p* < 0.05) whereas the M group showed a drastically lower relative abundance of Akkermansia than those of other groups. Of note, the relative abundance of Akkermansia was similar in the TS group and the M group. The relative abundance of Dubosiella was highest in the M group, followed by the C, TF, TG, and TS groups. We also found that the C group and the TS group had the highest relative abundance of Lachnospiraceae_NK4A136_group, which did not differ significantly between the two groups (*p* < 0.05). The M group displayed the lowest relative abundance of Lachnospiraceae_NK4A136_group, which was extremely significantly lower than those of the other groups (*p* < 0.01) (Figures 6C,D).

## Discussion

Generally, impaired glycolipid metabolism is a significant complication of T2DM (21), closely associated with high-calorie diets and obesity (22). Glycolipid metabolism can promote metabolic abnormalities and insulin resistance, leading to increased glucose production and defects in insulin secretion in the liver (23).

Our results showed that obese mice with T2DM had significantly increased BW and blood glucose levels (Figure 1), elevated serum FBG levels, as well as decreased INS (Figure 2). Insulin levels in the TF3, TG, and TS groups increased significantly when compared to the M group. The main reason is that high doses of GABA-rich sprouted adzuki beans, GABA, or metformin can depolarize islet cell membrane potential by activating GABA receptors (GABAbR) on the cell membrane, enhancing insulin secretion and maintaining glucose homeostasis. Of note, various dosages of GABA-rich sprouted adzuki beans can partially attenuate the symptoms of diabetes in the mice model. However, supplementation with high dosages of GABA-rich sprouted adzuki beans (TF3 group) exerted an excellent regulatory effect on blood glucose levels in mice with T2DM. The TF3 group showed 32.9 and 48.8% lower TG and TC serum levels than those in the M group, respectively (Figures 3A,B). High- dosages of GABA-rich sprouted adzuki beans also diminished the values of ALT and AST in mice serum with T2DM (Figures 3C,D). These data showed that high- dosages of GABA-rich sprouted adzuki beans supplementation has a positive effect on blood glucose homeostasis and alleviate pathological changes in liver and caecal tissues in mice with T2DM (Figures 4A,B). These results support prior reports that GABA-rich yogurt improves insulin resistance and HOMA-β of β-cells (16). Moreover, it has been shown that GABA can improve systemic insulin and increase glucose uptake in adipose tissue macrophages (24), thereby improving the maintenance of a balanced blood glucose level.

Here, we investigated the composition and richness of the gut microbiota in mice to explore the mechanism underlying the inhibitory effects of GABA-rich sprouted adzuki beans against HFD-induced T2DM. A preliminary phylum comparison revealed that the M group had a significantly higher density of Firmicutes and a significantly lower density of Bacteroidetes, with a Firmicutes/Bacteroidetes (F/B) ratio of 10.75, whereas mice in the TF groups had the lowest F/B ratio (Figure 5A). The results indicated that the dietary modification with GABA-rich sprouted adzuki beans significantly alters the frequencies of Firmicutes and Bacteroidetes, and the F/B ratio is positively associated with diabetes, consistent with previous findings (25, 26). A symbiotic relationship between Firmicutes and Bacteroidetes may regulate host metabolism by facilitating the intestinal absorption and storage of energy. Mice with T2DM in the M group displayed an elevated F/B ratio, which increased energy utilization. Mice in the intervention (TF, TG, and TS) groups had a lower F/B ratio with reduced energy absorption, thereby improving glucose metabolism disorders. Some studies have reported that increases in the abundances of *Clostridium difficile*, Eubacterium rectale, Verrucomi-crobiaceae, and other taxa can improve the symptoms of diabetes (27). In this study, intervention with TG and TF increased the relative abundance of Verrucomicrobia, which decreased to nearly zero in the M and TS groups, showed that the beneficial effect of high-dose GABA-rich sprouted adzuki beans (TF) on glycolipid metabolism is closely associated with the increase in the abundance of Verrucomicrobia. Peng et al. also confirmed that fecal metabolites are significantly affected by changes in the abundances of Verrucomicrobia and Bacteroidaceae (28), consistent with our findings. In addition, Lachnospiraceae NK4A136 and Akkermansia showed the most obvious upward trends in the TF treatment groups (Figure 5D), among which Akkermansia displayed the most significant increase in relative abundance, further demonstrating that high-dose GABA-rich sprouted adzuki beans intake can improve the gastrointestinal health of mice with T2DM, probably via the regulation of metabolism by boosting the number of beneficial bacteria in the intestines, so increasing the generation of short-chain fatty acids.

## Conclusion

In conclusion, our results showed that GABA-rich sprouted adzuki beans can effectively control the BW, maintain a balanced blood glucose level, improve glycolipid metabolism. Furthermore, GABA-rich sprouted adzuki beans can modify the microbiota composition in the caecum; in particular, it increased the relative abundances of Lachnospiraceae NK4A136 and Akkermansia to effectively improve glucose metabolism and gastrointestinal health. Ultimately, the host benefits from the regulatory effects of GABA-rich sprouted adzuki beans on blood glucose levels and metabolism. Our results provide a foundation for dietary intervention with highly processed GABA-containing beans for the treatment of T2DM. Then, using metabolomics and transcriptomics, we'll explore at the regulatory mechanism of sprouted adzuki beans on type 2 diabetes in the further, which is mediated by intestinal flora metabolites.

## Data Availability Statement

The original contributions presented in the study are included in the article/Supplementary Material, further inquiries can be directed to the corresponding author.

## Author Contributions

AZ: visualization, investigation, and validation. XJ: methodology, data analysis, drawing charts, and writing—original and editing. YG: conceptualization, methodology, and writing—review. QX: feeding animals and supporting partial data. ZL: conceptualization and funding acquisition. HT and DC: investigation and formal analysis. DZ: funding acquisition and supervision. All authors have read and agreed to the publishing of the current version of the manuscript.

## Funding

This work was supported by major science and technology special project of ten million project in Heilongjiang Province (2021ZX12B06), National Key R&D Plan, National Key R&D Plan of the Ministry of Science and Technology (2018YFE0206300), and Natural Science Fund Research Team Project of Heilongjiang Provincial Department of Science and Technology (TD2020C003).

## Conflict of Interest

The authors declare that the research was conducted in the absence of any commercial or financial relationships that could be construed as a potential conflict of interest.

## Publisher's Note

All claims expressed in this article are solely those of the authors and do not necessarily represent those of their affiliated organizations, or those of the publisher, the editors and the reviewers. Any product that may be evaluated in this article, or claim that may be made by its manufacturer, is not guaranteed or endorsed by the publisher.
