# Supplementary material for: The Effects of GABA-Rich Adzuki Beans on Glycolipid Metabolism, as Well as Intestinal Flora, in Type 2 Diabetic Mice
Source: Front Nutr. 2022 Feb 14;9:849529. doi: 10.3389/fnut.2022.849529 (PMC8883037; doi:10.3389/fnut.2022.849529)

**SUPPLEMENTARY FIGURE 1.** Experimental design of the 11-week dietary and pharmacological interventions for normal and HTD+STZ-induced T2DM mice

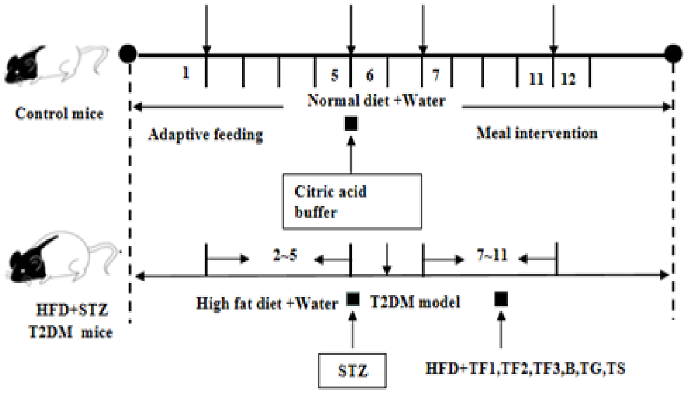

Supplement: Supplementary file 1 [file Image_1.pdf]
